# How Do CD4^+^ T Cells Detect and Eliminate Tumor Cells That Either Lack or Express MHC Class II Molecules?

**DOI:** 10.3389/fimmu.2014.00174

**Published:** 2014-04-15

**Authors:** Ole Audun Werner Haabeth, Anders Aune Tveita, Marte Fauskanger, Fredrik Schjesvold, Kristina Berg Lorvik, Peter O. Hofgaard, Hilde Omholt, Ludvig A. Munthe, Zlatko Dembic, Alexandre Corthay, Bjarne Bogen

**Affiliations:** ^1^Department of Immunology, Centre for Immune Regulation, Oslo University Hospital, University of Oslo, Oslo, Norway; ^2^KG Jebsen Centre for Research on Influenza Vaccines, Institute of Immunology, Oslo University Hospital, University of Oslo, Oslo, Norway; ^3^Faculty of Dentistry, Molecular Genetics Laboratory, Department of Oral Biology, University of Oslo, Oslo, Norway; ^4^Department of Biosciences, University of Oslo, Oslo, Norway; ^5^Tumor Immunology Group, Department of Pathology, Oslo University Hospital, University of Oslo, Oslo, Norway

**Keywords:** tumor immunology, CD4^+^ T cells, MHC class II, T cell receptor transgenic, transgenic mouse models, tumor antigen, T helper 1, multiple myeloma

## Abstract

CD4^+^ T cells contribute to tumor eradication, even in the absence of CD8^+^ T cells. Cytotoxic CD4^+^ T cells can directly kill MHC class II positive tumor cells. More surprisingly, CD4^+^ T cells can indirectly eliminate tumor cells that lack MHC class II expression. Here, we review the mechanisms of direct and indirect CD4^+^ T cell-mediated elimination of tumor cells. An emphasis is put on T cell receptor (TCR) transgenic models, where anti-tumor responses of naïve CD4^+^ T cells of defined specificity can be tracked. Some generalizations can tentatively be made. For both MHCII^POS^ and MHCII^NEG^ tumors, presentation of tumor-specific antigen by host antigen-presenting cells (APCs) appears to be required for CD4^+^ T cell priming. This has been extensively studied in a myeloma model (MOPC315), where host APCs in tumor-draining lymph nodes are primed with secreted tumor antigen. Upon antigen recognition, naïve CD4^+^ T cells differentiate into Th1 cells and migrate to the tumor. At the tumor site, the mechanisms for elimination of MHCII^POS^ and MHCII^NEG^ tumor cells differ. In a TCR-transgenic B16 melanoma model, MHCII^POS^ melanoma cells are directly killed by cytotoxic CD4^+^ T cells in a perforin/granzyme B-dependent manner. By contrast, MHCII^NEG^ myeloma cells are killed by IFN-γ stimulated M1-like macrophages. In summary, while the priming phase of CD4^+^ T cells appears similar for MHCII^POS^ and MHCII^NEG^ tumors, the killing mechanisms are different. Unresolved issues and directions for future research are addressed.

## Recent Advances in Tumor Immunology

The field of tumor immunology has come a long way since the formulation of the tumor immunosurveillance hypothesis by Thomas and Burnet (1–4). Although still debated, increasing evidence suggests that the immune system can detect and reject incipient tumors, and that CD4^+^ and CD8^+^ T cells play an important role as mediators of immunosurveillance (5). Furthermore, there is accumulating evidence that the immune system is not completely tolerant even to established tumors, based on the observation that tumor-infiltrating T cells, when expanded *in vitro* and injected back to lymphopenic patients, have a clinical effect in some patients (6). Further supporting the notion of ongoing immune responses to tumors, antibodies that block inhibitory molecules on T cells induce long-term remission in a subset of cancer patients (7). Finally, parameters that indicate immune activation in tumors are associated with improved prognosis (8).

## CD4^+^ versus CD8^+^ T Cells in Tumor Immunology

Traditionally, CD8^+^ T cells have been thought to be the major mediators of effective anti-tumor T cell responses. Such a view is supported by the pronounced cytotoxic activity of CD8^+^ T cells *in vitro*, and the observation that tumors that escape CD8^+^ T cells onslaught may have altered or downregulated MHC class I antigen expression (9–11). Moreover, studies done in an MHC class I-restricted T cell receptor (TCR) transgenic mouse showed that CD8^+^ T cells, in the absence of CD4^+^ T cells, maintained their anti-tumor effect (12). Despite these observations, several studies indicate limited anti-tumor effects of CD8^+^ T cells alone (6, 13–16).

The helper function of tumor-reactive CD4^+^ T cells improves the efficacy of tumor-reactive CD8^+^ T cells (17–20). Similarly, treatment of a patient with metastatic melanoma with autologous CD4^+^ T cells specific for the tumor-associated antigen NY-ESO-1 resulted in sustained clinical remissions with evidence of endogenous immune responses against other tumor-derived antigens (21). In support of these findings, transfection of tumor cells with MHC class II genes resulted in increased protective immune responses against tumors (22, 23). Collectively, these results indicate an augmenting effect of CD4^+^ T cells on CD8^+^ T cell responses against tumors.

On the other hand, CD4^+^ T cells alone, in the absence of CD8^+^ T cells, have also been demonstrated to eliminate tumor cells. Thus, adoptive transfer experiments using primed CD4^+^ T cells generated by immunization with tumor cells conferred protection against a subsequent tumor challenge (24, 25). Moreover, naïve CD4^+^ T cells in TCR-transgenic mice conferred protection against tumor development upon subcutaneous (s.c.) injection of tumor cells (26, 27). Finally, using MHC class I-molecule and MHC class II-molecule restricted TCR-transgenic mice specific for the Dby H–Y antigen, CD4^+^ T cells were found to be more efficient at eradicating cancer cells than CD8^+^ T cells in a side-by-side comparison (28). Here, we will focus on the anti-tumor properties of CD4^+^ T cells in the absence of CD8^+^ T cells.

## Pioneering Experiments on the Role of CD4^+^ T Cells in Eradication of Tumors

The role of CD4^+^ T cells was initially investigated in experiments where tumor-bearing mice were treated by adoptive transfer of T cells obtained from syngeneic mice immunized with irradiated tumor cells (25, 29), or with living tumor cells followed by surgical resection (24). It was shown that when T cells from tumor-immunized donors were purified prior to adoptive transfer, Lyt1^+^ 2^−^ (CD4^+^) T cells had a superior ability to cure FBL-3 erythroleukemic tumors compared to Lyt1^−^ 2^+^ (CD8^+^) T cells (29). Treatment with cyclophosphamide was required for the curative effect of CD4^+^ T cells to be observed. However, in the first reported experiments (29), a role of endogenous CD8^+^ T cells in the tumor-bearing host was not ruled out. In follow-up experiments, this possibility was formally excluded by the use of T cell deficient tumor-bearing recipients (25). Similar results were obtained using the X5563 plasmacytoma model (24), where transfer of purified Lyt1^+^ 2^−^ (CD4^+^) T cells had superior therapeutic potential. In the following decades, experimental evidence supporting the anti-tumor properties of tumor-specific CD4^+^ T cells alone has accumulated (27, 28, 30–39).

## TCR-Transgenic Models for CD4^+^ T Cell-Mediated Rejection of Tumors

The experiments referred to in the preceding section had features that prohibited detailed studies of the mechanisms of CD4^+^ T cell-mediated tumor protection. First, the CD4^+^ T cells were polyclonal. Second, CD4^+^ T cells were pre-primed cells obtained after immunization, making it impossible to study naïve CD4^+^ T cells in primary anti-tumor responses. Third, the relevant tumor-specific antigens were often not known.

The generation of TCR-transgenic mice that recognize tumor antigens presented on MHC class II molecules (Table 1) offered a novel approach to bypass these difficulties. In two models, these antigens are *bona fide* cancer antigens; the tumor-specific myeloma protein V region idiotype (Id) (26, 27) and the melanoma-associated tyrosinase-related protein 1 (Trp1) (35). In other TCR-transgenic models, the antigens are either minor histocompatibility antigen Dby (H-Y) (28), viral antigens such as the hemagglutinin (HA) (40–42), or xenogeneic proteins such as ovalbumin (OVA) (17, 43, 44). While the transgenic TCR specific for the mutated myeloma antigen was obtained after immunization of mice syngeneic to the tumor (45, 46), the transgenic TCR specific for the non-mutated antigen was obtained after immunization of Trp1-deficient mice. Thus, in the latter model, Trp1 represents a foreign antigen to which high-affinity TCRs are induced (due to a lack of T cell tolerance) (35).

**Table 1:** **TCR-transgenic models employed in studies of anti-tumor CD4^+^ T cell responses**.

TCR-Tg model	Antigen	Classification of antigen	Antigen location	MHC II restriction	Peptide	Reference
4B2A1 (λ2^315^)	Light chain idiotype (Id) of mouse M315 myeloma protein	Mutated tumor-specific antigen	Secreted, plasma membrane (52, 53)	I–E^d^	aa91–101	(46)
7A6 (Trp1)	Mouse tyrosinase-related protein 1	Melanocyte-specific differentiation antigen	Secreted, melanosome membrane (54)	I–A^b^	aa113–125	(35)
Marilyn (H–Y)	Minor histocompatibility antigen (Dby)	Tissue antigen	Secreted, cell membrane (55, 56)	I–A^b^	aa608–622	(47)
T2.5-5 (HA)	Influenza PR8 hemagglutinin	Viral antigen	Varying (construct dependent)^1^	I–A^d^	aa126–138	(48)
14.3.d (HA)	Influenza PR8 hemagglutinin	Viral antigen	Varying (construct dependent)^1^	I–E^d^	aa110–120	(49)
DO11.10 (OVA)	Chicken ovalbumin	Xenogeneic model antigen	Varying (construct dependent)^2^	I–A^d^	aa323–339	(50)
OT-II (OVA)	Chicken ovalbumin	Xenogeneic model antigen	Varying (construct dependent)^2^	I–A^b^	aa323–339	(51)

*^1^Varyingly expressed by fusion to other proteins, which control cellular distribution. The viral protein, as such, localizes to the cell surface (57)*.

*^2^Varyingly expressed as full-length cDNA [containing signal sequence for secretion (58)] or fused to other proteins, which control cellular distribution*.

## MHC Class II Status of Tumor Cells Used in Tumor Immunology Studies Focused on the Role of CD4^+^ T Cells

CD4^+^ T cells recognize peptides (about 13–17aa long) bound to the groove of MHC class II molecules (59) on professional antigen-presenting cells (APCs) (B cells, dendritic cells, macrophages, in addition to thymic epithelial cells) (60–62). However, in certain cells, MHC class II molecules may be induced by interferon gamma (IFN-γ) stimulation (63, 64). Thus, in CD4^+^ T cell immune responses to tumors, the MHC class II status of the tumor cells is of importance. The MHC II expression status of tumor cells used in studies with CD4^+^ TCR-transgenic mice is summarized in Table 2.

**Table 2:** **Use of TCR-Tg models for studies of anti-tumor CD4^+^ T cell immune responses**.

TCR-Tg model (antigen)	Tumor cell line	Ectopic antigen expr.^a^	MHC II expr.	Antigen secreted?	T cell source	Reference
4B2A1 (λ2^315^)	MOPC315 (plasmacytoma)	No	−	Yes	Naïve (endogenous)^b^	(26, 27, 34, 65)
	MOPC315.37^c^	No	−	No	Naive (endogenous)	(36)
	A20 (B lymphoma)	Yes	+	Yes	Naive (endogenous)	(26, 33, 66)
					Adoptive transfer, naive	
	A20 (B lymphoma)^d^	Yes	+	No	Naive (endogenous)	(26)
7A6 (Trp1)	B16/CIITA (melanoma)	No	+^e^	N/D	Naive (endogenous)	(35)
					Adoptive transfer, activated	
	B16 (melanoma)	No	+^f^	N/D	Adoptive transfer, naïve	(37, 38)
					Adoptive transfer, activated	
Marilyn (H–Y)	MB49 (bladder)	No	+^f^	N/D	Adoptive transfer, naive	(28)
	TRAMP-C2 (prostate)	No	−	N/D	Adoptive transfer, activated	
	βTC-TET	No	−	N/D		
	WR21 (salivary gland)	No	−	N/D	
T2.5-5 (HA)	AB1 (mesothelioma)	Yes	−	N/D^g^	Naive (endogenous)	(40)
					Adoptive transfer, naive	
14.3d (HA)	CT26 (colon)	Yes	N/D^h^	N/D^i^	Naive (endogenous)	(41, 42)
					Adoptive transfer, naive	
DO11.10 (OVA)	A20 (B lymphoma)	Yes	+	N/D^j^	Adoptive transfer, activated	(17)
	A20 (B lymphoma)	Yes	+	No^k^	Naive (endogenous)	(44)
					Adoptive transfer, activated	
OT-II (OVA)	EG-7 (thymoma)	Yes	−	Yes^l^	Adoptive transfer, activated	(43)

*N/D, not determined*.

*^a^Ectopic antigen expression signifies that the tumor cell line was transfected for expression of the relevant antigen*.

*^b^The designation naive (endogenous) is used to describe tumor challenge experiments in TCR-Tg mice in which no prior priming of antigen-specific T cells was performed*.

*^c^MOPC315.37 contains a Gly15 → Arg15 mutation within the λ2 gene that causes intracellular retention (67)*.

*^d^Cells were transfected with a mutated λ2^315^ variant that causes retention within the endoplasmic reticulum, precluding secretion (67)*.

*^e^Cells were transfected to overexpress MHC class II trans-activator (CIITA) to ensure high levels of expression of MHC II (35)*.

*^f^Inducible expression by interferon gamma stimulation*.

*^g^Only cell surface expression was tested (40)*.

*^h^A previous publication reports constitutive MHC II expression *in vitro* (68)*.

*^i^Cells were transfected with HA fused to EGFP. Only surface expression was tested (41)*.

*^j^Secretion expected; cells were transduced with constructs containing the full-length OVA cDNA sequence, which contains signal element for secretion (58)*.

*^k^Cells were transfected with OVA fused to the trans-membrane domain of transferrin receptor, causing membrane expression (44)*.

*^l^Earlier report demonstrates secretion from the same cell line (69)*.

## Direct and Indirect Killing of Tumor Cells by CD4^+^ T Cells

**Figure 1:**
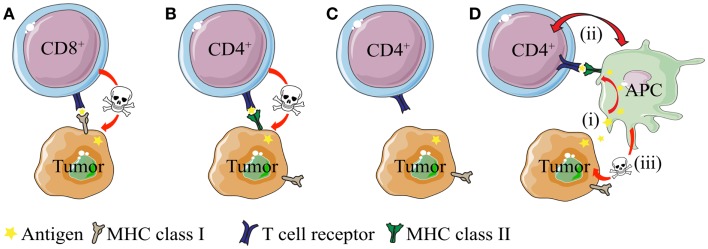
**Direct and indirect killing of tumor cells by CD4^+^ T cells**. **(A)** CD8^+^ T cells can directly kill tumor cells that express MHC class I molecules, whereas **(B)** cytotoxic CD4^+^ T cells can kill tumor cells that express MHC class II molecules. **(C)** While most tumor types express MHC class I molecules, they often lack expression of MHC class II. How do CD4^+^ T cells recognize and eliminate MHCII^NEG^ tumor cells? **(D)** CD4^+^ T cells may kill MHC class II negative (MHC II^NEG^) tumors by a mechanism where (i) tumor antigen secreted by tumor cells is processed and presented by MHCII^POS^ macrophages to CD4^+^ T cells. (ii) Bi-directional interaction/activation of macrophages and CD4^+^ T cells (iii) activates tumoricidal macrophages that in turn kill the tumor cells (In addition, activated CD4^+^ T cells themselves could possibly directly kill tumor cell in a TCR/MHC II-independent manner.).

The antigen-specific interaction between CD4^+^ T cells and MHC II^POS^ tumor cells is conceptually easy to grasp. On the other hand, the basis for antigen presentation and anti-tumor effector mechanisms are less obvious in the context of MHC II^NEG^ tumors (25, 26, 31, 70) – simply because such cancer cells cannot directly stimulate MHC class II-restricted CD4^+^ T cells (Figure 1). In the following sections, we discuss mechanism of CD4^+^ T cell-mediated direct killing of MHC II^POS^ tumor cells and indirect killing of MHC II^NEG^ tumor cells. Emphasis is put on observations from TCR-transgenic models, where the T cell specificity is known and both naïve and primed CD4^+^ T cells are readily available.

## Direct Killing of MHC Class II^POS^ Tumor Cells

The existence of CD4^+^ T cells with cytotoxic properties has been increasingly recognized throughout the last three decades. Such cells are thought to function in a fashion analogous to cytotoxic CD8^+^ T cells, with antigen recognition triggering the release of cytotoxic mediators. CD4^+^ T cells displaying direct cytotoxicity *in vitro* toward MHC II^POS^ targets, including tumor cells, have been described by several authors (37, 45, 70, 71). Correspondingly, efficient elimination of MHC II^POS^ tumors by T cells with such properties is also observed *in vivo* (26, 28, 33, 35, 37, 38, 72).

Several effector mechanisms have been implicated for tumor-specific cytotoxic CD4^+^ T cells. In a model of Id-specific CD4^+^ T cell responses against an MHC II^POS^ B lymphoma, *in vitro* cytotoxicity was shown to be dependent on signaling mediated by binding of Fas ligand (FasL) on CD4^+^ T cells to the death receptor Fas on tumor cells (66). Naïve T cells showed little killing activity, whereas Th1 differentiation greatly enhanced cytotoxicity. However, *in vivo* elimination of tumor cells was not affected in FasL-deficient (*gld*^−/−^) Id-specific TCR-Tg mice, suggesting that signaling through the Fas pathway is dispensable for tumor killing and that additional mechanisms are operational *in vivo* (66). Indeed, if the tumor antigen is secreted as is the case in the studies of Lundin et al. (33, 66), the indirect mechanism via Th1/M1 macrophages described below could also be active, and might play a prominent role in tumor rejection. In the Trp1-specific TCR-transgenic model, it was demonstrated that the rejection of B16 melanoma cells was abrogated in mice deficient for either granzyme B or perforin, indicating that these molecules are important for CD4^+^ T cell-mediated killing of MHC II^POS^ tumor cells (37). In summary, different MHC II^POS^ tumors may vary in susceptibility to various effector mechanisms of CD4^+^ T cells, as indicated by the observations addressed above.

## Indirect Killing of MHC Class II^NEG^ Tumor Cells

In general, antibody-secreting plasma cells are MHC class II negative due to silencing of the MHC Class II trans-activator (CIITA) occurring during plasma cell differentiation (73, 74). Multiple myeloma (MM) is the malignant counterpart of plasma cells and usually express little if any MHC class II molecules. MHC class II negativity due to loss of CIITA expression appears to be a stable phenotype, although some studies have reported MHC II upregulation in MM cells exposed to retinoic acid (75) or IFN-γ (76, 77).

**Figure 2:**
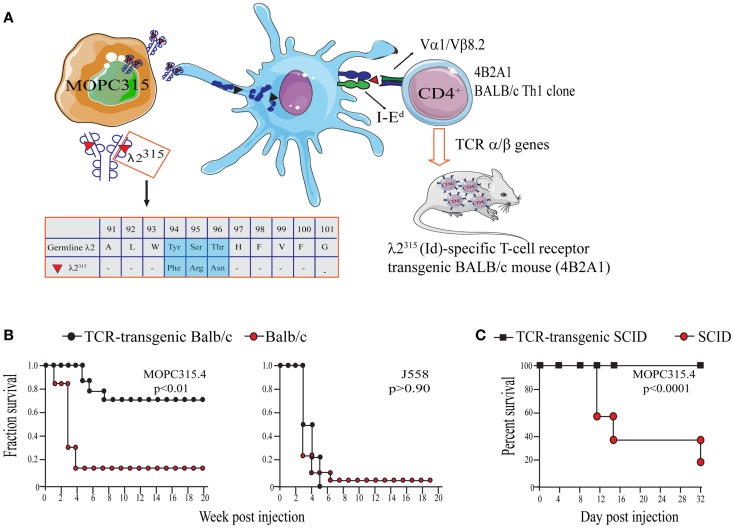
**The MOPC315 myeloma model**. Naïve tumor-specific CD4^+^ T cells protect against MHC II^NEG^ tumor challenge in the absence of other T cells and B cells. **(A)** MOPC315 myeloma cells of BALB/c origin secrete an IgA M315 myeloma protein with a mutated λ2 light chain referred to as λ2^315^. M315 is endocytosed and processed by BALB/c APCs, and a CDR3 sequence that includes residues 91–101 of λ2^315^ is presented on the MHC class II molecule I-E^d^ to Id-specific CD4^+^ T cells. The peptide that is recognized by Id-specific CD4^+^ T cells contains somatic mutations in positions 94, 95, and 96 (45, 79, 80). Based on the αβ TCR of the Id-recognizing 4B2A1 clone, a TCR-transgenic mouse was generated (46). Most CD4^+^ T cells in this mouse express a transgenic TCR that can be tracked by a clonotype-specific mAb [Nomenclature: antigenic determinants in immunoglobulin variable (V) regions are called idiotopes (Id). The 91–101 peptide is thus an Id-peptide, and the CD4^+^ T cells that recognize this Id-peptide presented by I-E^d^ are called Id-specific]. **(B)** Id-specific TCR-transgenic mice on an immunosufficient background (BALB/c) are resistant to a challenge with Id^POS^ MOPC315 cells but succumb to Id^NEG^ J558 myeloma cells [reproduced with permission from Proc Natl Acad Sci (26), Copyright 1994 National Academy of Sciences, U.S.A.]. **(C)**. Id-specific TCR-transgenic mice on an immunodeficient background (SCID), lacking other T and B cells than Id-specific CD4^+^ T cells, are also resistant to MOPC315 tumor development [reproduced with permission from Immunity (34)]. Tumor resistance could be transferred with purified Id-specific CD4^+^ T cells to SCID mice (27).

The work of our research group is based on experiments using the mineral oil-induced BALB/c plasmacytoma (MOPC)315 (52, 70). MOPC315 cells secrete a highly mutated and unique monoclonal IgA (myeloma protein). The λ2 light chain of the myeloma protein contains somatic mutations in positions 38, 50, 94, 95, and 96 that are unique to MOPC315 (78). Thus, the myeloma protein light chain is referred to as λ2^315^ (Figure 2A).

By immunization of BALB/c mice with free λ2^315^ L chain, known from previous studies to stimulate T cells (81), I-E^d^-restricted, Id-specific CD4^+^ T cell clones were generated (Figure 2A) (45). These clones recognize a unique Id-epitope, which depends on the somatic mutations in codons 94, 95, and 96 within the CDR3 loop of the λ2^315^ light chain (79). As would be expected, MOPC315 derived λ2^315^-immunoglobulin has to be endocytosed and processed by APCs prior to MHC class II presentation of the Id-peptide (80).

MOPC315 is found to be MHC class II negative by a number of criteria: (i) Negative staining with anti-MHC class II antibodies both *in vitro, ex vivo* (70), and *in vivo* (65). Lack of expression of MHC II molecules on MOPC315 was independently reported by others (82). (ii) Exposure to high amounts (500 ng/ml) of IFN-γ IL-4, or supernatant from activated Th1 cells, all failed to induce any detectable expression of MHC class II *in vitro* (70). (iii) Both *in vitro*-cultured (70) and *ex vivo* (65) MOPC315 cells failed to stimulate Id-specific MHC class II-restricted T cells in proliferation and cytokine secretion assays.

## Idiotype-Specific CD4^+^ T Cell Clones Induce Killing of MHC Class II Negative Myeloma Cells *in vitro* – but Only in the Presence of MHC-Compatible APCs

A weak cytotoxicity that was greatly augmented by addition of high amounts of myeloma protein was observed when Id-specific CD4^+^ T cells were co-cultured with MHC-compatible spleen cells from BALB/c (H-2^d^) MHC II^NEG^ MOPC315. Importantly, MHC II incompatible spleen cells from C57BL/6 failed to support cytotoxicity (70). Moreover, the cytotoxic effect could not be transferred by supernatants of activated T cells. It was suggested that some of the spleen cells, e.g., macrophages (Mϕ) stimulated by activated T cells, were important as cytotoxic effector cells in the *in vitro* cultures (70).

## Naïve Id-Specific CD4^+^ T Cells in T Cell Receptor Transgenic Mice Protect Against Id^+^ Myeloma Cells in the Absence of CD8^+^ T Cells and B Cells

To facilitate studies of the role of Id-specific CD4^+^ T cells in tumor protection against MHC II negative MOPC315, an Id-specific TCR-transgenic mouse on syngeneic BALB/c background was established (46).

In initial experiments, naïve Id-specific T cells from TCR-transgenic mice did not respond to MOPC315 *in vitro*. Despite this, Id-specific TCR-transgenic mice were specifically protected against s.c. challenge with MOPC315 cells (26) (Figure 2B). Eradication of MOPC315 cells resulted in a change of T cell phenotype, since T cells of surviving TCR-transgenic mice had increased cytotoxicity against Id^+^ MHC II^POS^ B lymphomas, and since they upon stimulation produced much IFNγ and some IL-4.

By breeding the TCR-Tg mice onto a SCID background, it was demonstrated that rejection of MOPC315 was independent of CD8^+^ T cells and B cells/antibodies (27, 34) (Figure 2C). Additionally, tumor protection could be transferred to SCID mice with adoptive transfer of purified Id-specific CD4^+^ T cells (27).

## Id-Primed APC Can be Detected in Tumor Tissue of Large Established Myelomas

The finding that naïve CD4^+^ T cells could initiate rejection of a MHC II negative tumor indicated that host cells expressing MHC class II molecules were responsible for the presentation of Id to CD4^+^ T cells. In a subsequent study, it was demonstrated that s.c. MOPC315 tumors contained APCs that were able to stimulate Id-specific CD4^+^ T cells *in vitro* in an MHC-restricted manner (65). The great majority of MHC II^POS^ tumor-infiltrating APCs were CD11b^+^CD11c^LOW^CD80^+^CD86^+^. These studies demonstrated that MHC class II negative MOPC315 tumors were infiltrated with Id-primed APCs with macrophage-like characteristics.

## Id-Specific CD4^+^ T Cells are Present and Activated in Tumor Tissue

Given that Id-primed APC could be demonstrated in MOPC315 tumors, it was investigated if Id-specific CD4^+^ T cells were also present, and whether they were activated. In these experiments, a high amount of MOPC315 cells were injected in order to overcome the resistance of TCR-transgenic mice. A number of observations indicated that Id-specific CD4^+^ T cells were specifically activated in small s.c. MOPC315 tumors established in Id-specific TCR-transgenic mice: (i) The CD4^+^/CD8^+^ ratio was skewed toward CD4^+^ in tumor tissue. (ii) CD4^+^ blasts within the tumor were selectively enriched for cells expressing the Id-specific TCR. (iii) Id-specific CD4^+^ tumor-infiltrating lymphocytes (TIL) were activated (CD69^+^ CD25^+^), and proliferated (BrdU^+^) in clusters associated with MHC II^POS^ tumor-infiltrating APC (65).

## Secretion of Tumor-Specific Antigen is Required for CD4^+^ T Cell-Mediated Rejection of MHC II^NEG^ Tumors

While it was clear that tumor-infiltrating APCs and lymph node cells take up the λ2^315^ antigen and display the Id-peptide on MHC class II molecules (34, 65), the precise source of the priming Id antigen was not established. To address this question, we used two secretory variants of MOPC315: one that secretes the complete M315 myeloma protein composed of α H chain and λ2^315^ L chain (MOPC315), and another that only secrets the free λ2^315^ L chain (MOPC315.26). In addition, we used two non-secretory variants: one where the free λ2^315^ L chain is retained intracellularly due to a point mutation (MOPC315.37) and another where no Ig is produced (MOPC315.36) (67, 83).

When Id-specific TCR-transgenic SCID mice were challenged with the four variants, protection was observed for the λ2^315^-secreting variants MOPC315 and MOPC315.26, while there was no protection against the antigen-negative MOPC315.36. Tumor take was significantly delayed, but still complete, in mice challenged with the MOPC315.37, which retains λ2^315^ intracellularly. This result was surprising since in MOPC315.37-containing Matrigels, macrophages were MHC II^HI^, and Id-specific T cells were activated (CD69^+^). The only striking deficiency observed with MOPC315.37 *in vivo* was deficient T cell activation in draining lymph nodes, presumably due to poor local availability of the intracellularly retained tumor antigen. These results indicate that the extracellular concentration of secreted tumor-specific antigen is important for protection against an MHC II^NEG^ tumor, most likely due to enhanced priming of APCs in draining lymph nodes as well as macrophages in tumors (36, 84).

## Detection of Tumor-Specific CD4^+^ T Cells and Macrophages in Early Stages after Tumor Cell Challenge: The Matrigel Method

**Figure 3:**
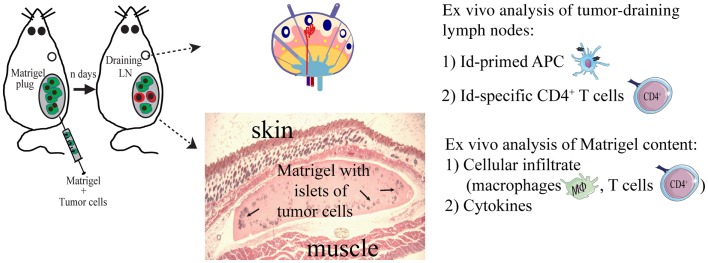
**The Matrigel assay**. A novel approach to unravel the dynamics of CD4^+^ T cell-mediated primary anti-tumor immune responses. At day 0, subcutaneous injections with MOPC315 tumor cells suspended in liquid Matrigel. When the Matrigel solution reaches body temperature, it jellifies and forms a plug containing the tumor cells. At various time points after injection (*n* days), the Matrigel plug and tumor-draining lymph nodes are dissected out and analyzed *ex vivo* for cellular content, function of cells, and cytokines (34, 36, 39, 85).

**Figure 4:**
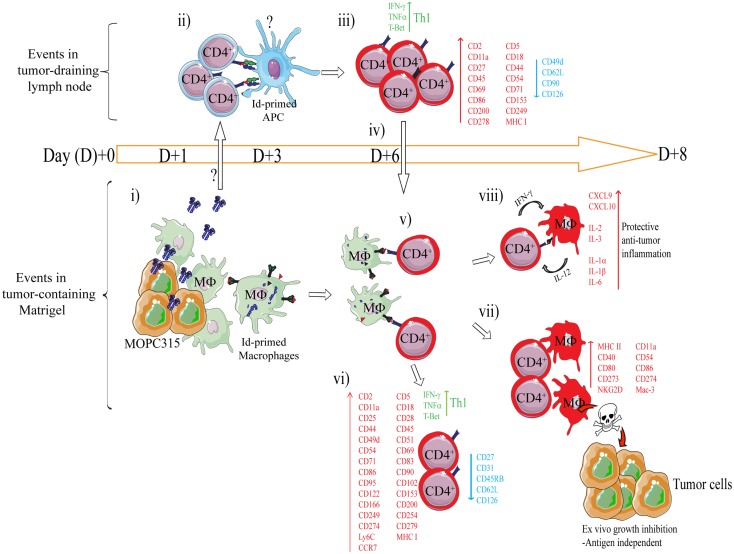
**Mechanism of rejection of MHCII^NEG^ myeloma cells by Id-specific CD4^+^ T cells**. The following events are based on experiments where Id-secreting MOPC315 suspended in liquid Matrigel was injected subcutaneously in TCR-transgenic mice. (i–viii). (i) At the incipient tumor site, macrophages [CD11b^+^, CD11c^−^, CD80/CD86^+^ MHC II^LO^, F4/80^+^] start to infiltrate the tumor/Matrigel from day +1. Tumor-infiltrating macrophages become Id-primed by extracellular myeloma protein by the conventional MHC II presentation pathway (65). (ii) Extracellular Id+ myeloma protein (or possibly Id-primed tumor APCs) drain to lymph nodes where Id-primed APCs stimulate Id-specific CD4^+^ T cells. Uncertainties as to the mechanism of Id+ Ag draining and the identity of Id-primed APCs are indicated by a question mark (?). (iii) Id-specific CD4^+^ T cells become activated by day +3, are substantially expanded by day +6 (34), and polarize into Th1 cells by day +8 (39, 85). Upon activation in the tumor-draining lymph node, a number of molecules are significantly upregulated on the surface of the Id-specific CD4^+^ T cells, while some are consistently downregulated (85). (iv) Activated CD4^+^ T cells (CD62L^LOW^) leave the lymph node and accumulate at the tumor site from day +6 (34, 86). (v) At the incipient tumor site, infiltrating Id-specific CD4^+^ T cells are re-activated by Id-primed macrophages (34). (vi) Moreover, in addition to a sustained Th1 phenotype, the tumor-infiltrating CD4^+^ T cells dramatically change expression of a number of surface molecules (85). Several molecules are upregulated on both activated CD4^+^ T cells in the tumor-draining lymph node, and on tumor-infiltrating CD4^+^ T cells, although at higher levels in the latter population. (vii) IFN-γ produced by tumor-infiltrating Th1 cells activates macrophages that up-regulate MHC class II on the cell surface and show increased expression of M1-associated surface molecules (34, 39). IFN-γ-activated macrophages acquire a tumoricidal phenotype with the upregulation of cytotoxicity-associated markers including granzyme A/B, and NKG2D (39). In addition, purified activated macrophages can directly inhibit myeloma growth *in vitro* (34, 36, 39). The mechanisms underlying M1 macrophage-mediated growth inhibition is unknown, but once the macrophages are activated the growth inhibition is antigen independent (36). (viii) Analysis by gene expression profiling and Luminex multiplex cytokine analyses has revealed that the Id-specific CD4^+^ Th1-mediated anti-tumor immune response has a striking resemblance to the characteristics of acute inflammatory responses (39). Thus, we propose that Th1-mediated inflammatory responses may protect against cancer (87).

To study local events at the injection site at the early stages of the anti-tumor immune response, we injected the tumor cells suspended in a Matrigel solution (Figures 3 and 4). Matrigel is a liquid basement membrane preparation that jellifies rapidly at body temperature. Thus, a tumor bed of a defined size was generated that could be isolated and assayed to characterize infiltrating cells at any time point following tumor cell injection (Figure 3). Moreover, the defined volume of the gel plug allows quantitative assays of secreted factors within the tumor microenvironment (39). Initial experiments demonstrated that tumor cells embedded in Matrigel were rejected by TCR-transgenic SCID mice, although less efficiently than in the absence of Matrigel (34). Thus, events in the tumor cell-containing Matrigel most likely reflected those taking place during successful immunosurveillance of MHC II negative tumor cells by CD4^+^ T cells.

Using this system, a longitudinal characterization of the immune response within the tumor microenvironment and draining lymph nodes was undertaken (34, 36, 39, 85). The findings are summarized in Figure 4. Briefly, secreted myeloma protein is presented by APC in tumor-draining lymph nodes to Id-specific CD4^+^ T cells. Upon recognition, T cells are activated, polarize into Th1 cells, and migrate to the Matrigel/tumor. In the Matrigel/tumor, Th1 cells become re-activated by tumor-infiltrating macrophages that has endocytosed and processed myeloma protein. Th1 derived IFN-γ activates macrophages into tumoricidal M1 macrophages (34, 36, 39, 65).

## Unresolved Issues and Directions for Future Research

### Use of MHC class II negative tumor cell lines in tumor immunology

While MHC class II positivity in tumor cells is generally to be trusted, MHC class II negativity should, for obvious reasons, be viewed with a healthy skepticism. In the case of MOPC315, many attempts by others and us have consistently failed to detect expression of MHC class II molecules *in vitro* as well as *in vivo*, even when MOPC315 cells were exposed to IFN-γ (34, 70, 82, 88). In several other models, such as the use of the erythroleukemia cell line FBL-3 (25), the UV-induced fibrosarcoma 6132A-PRO (31), and the methylcholanthrene-induced Mc51.9 (32); no MHC class II was detected on tumor cells even after IFN-γ exposure, similar to MOPC315. In the B16 melanoma model, Quezada et al. showed that the cell line used in their experiments expressed MHC class II, but only when the tumor-bearing hosts were subjected to a combination of irradiation and adoptive transfer of Trp1-specific CD4^+^ T cells together with anti-CTLA mAb (37). Xie et al. also reported that B16 cells express MHC class II by immunofluorescence staining of tumor biopsies, but the identity of the MHC class II^POS^ cells within the sections was not further characterized, complicating interpretation (38). In contrast, Hung et al. reports the use of B16 tumor cells that were described to be MHC II^NEG^ (30).

Peres-Diez et al. (28) reported that expression of MHC class II molecules on tumor cells was not required for rejection mediated by CD4^+^ cells. In note, they found that: H-2^k^ H-Y^+^ tumor cells were rejected by I–A^b^-restricted, H–Y-specific CD4^+^ T cells in an immunodeficient H-2^b^ mouse. An alternative approach to ensure the absence of the relevant MHC class II molecule in a tumor cell line would be to delete the corresponding MHC class II molecule genes from the tumor cells.

### Is secretion of tumor-specific antigen by MHC II^NEG^ tumors required?

The tumor-specific antigen used in our own studies, the MOPC315 myeloma protein, is a highly secreted antigen, with serum levels reaching milligrams per milliliter levels. Concentrations of myeloma protein in tumor tissues would be expected to be even higher. Surprisingly, a non-secreting myeloma variant that only expresses an intracellularly retained mutated Id^+^ L chain, but in high amounts, was not rejected (36). In the absence of sufficient tumor antigen secretion, it might be expected that either spontaneous necrosis or apoptosis of tumor cells containing such high amounts of intracellular tumor antigen could prime tumor-infiltrating APC with tumor-specific antigen. This is apparently not the case for the non-secreting variant of MOPC315. It remains to be seen whether cytotoxic drug treatment of mice with tumors caused by this particular MOPC315 variant could enhance Id priming of APCs via uptake of necrotic or apoptotic cells.

In other MHC II^NEG^ models where tumor cells is reported to be rejected by CD4^+^ T cells (28, 31, 32), there is scarce information as to whether tumor-specific antigen is secreted or not (Table 2). In the case of H-Y antigen, which clearly must be transferred from the tumor cells to host APC for MHC II presentation (28), there is little information about the extent of secretion of the antigen. In yet other cases, the tumor-specific antigen is simply not known (25), precluding any analysis of secretion status. It should further be noted that in some experiments [e.g., Ref. (24, 25, 37)] it has not been rigorously excluded that non-malignant normal cells could also produce the “tumor-specific” antigen. This possibility is virtually excluded in the MOPC315 model since CD4^+^ T cells recognize a somatically mutated tumor-specific antigen unique to MOPC315 myeloma cells. By and large, it appears that secretion of tumor-specific antigen facilitates priming of host APC and stimulation of CD4^+^ T cells. However, it is possible that the requirement of secretion could vary for distinct tumors and tumor-specific antigens, perhaps related to differences in susceptibility for cross-presentation of antigen associated with either necrotic or apoptotic tumor cells, or secreted vesicles such as exosomes.

What about MHC II^POS^ tumors – do they also require secretion of tumor-specific antigen? For MHC II^POS^ B lymphoma, a transfectant that secretes λ2^315^ was rejected, while another transfectant expressing a mutated intracellularly retained λ2^315^ was not (26). Similarly, A20 cells expressing HA, which apparently was negligibly secreted since HA was not found in serum, was not rejected (89). The Dby minor histocompatibility antigen (H–Y) (28) and Trp1 (35, 37, 38) have both been reported to be secreted by tumor cells. A strategy to test the hypothesis that secretion of tumor-specific antigen is required for rejection of MHC II^POS^ tumors would be to transfect MOPC315.37 with CIIITA so that the tumor cells become MHC II^POS^. If this transfectant is rejected in Id-specific TCR-transgenic mice, this would weaken the hypothesis.

### By which pathway is tumor antigen presented by APC in draining lymph nodes?

In the tumor models where it has been tested, be they MHC II^NEG^ (28, 34, 65) or MHC II^POS^ (37, 38), there was an apparent need for tumor-specific antigen to be presented by host APC to stimulate naïve (but not memory) CD4^+^ T cells. Thus, in the case of the B16 MHC II^POS^ model, no rejection by naïve Trp1-specific CD4^+^ T cells was obtained in hosts that lacked MHC class II molecules. By contrast, transfer of CD4^+^ T cells that first had been primed *in vitro* could readily reject B16 tumors (37, 38). These findings indicate that MHC II^POS^ tumor cells themselves are incapable of stimulating naïve Trp1-specific CD4^+^ T cells, and that priming by professional host APC is required. In addition, experiments reported by Xie et al. (38) using Trp1-deficient mice indicate that Trp1 derived from host tissue is redundant for priming APC and that Trp1 derived from B16 tumor cells suffice, at least for stimulation of memory CD4^+^ T cells. It is still, however, unclear how the Trp1 antigen is transferred from tumors to host APC, and in which anatomical compartment priming of CD4^+^ T cells take place.

The conclusions of the above experiments are supported by previous observations in the MOPC315 model, which directly demonstrate activation of Id-specific CD4^+^ T cells in draining lymph nodes (34, 36, 85). Moreover, treatment with the sphingosine phosphate receptor modulator fingolimod that abrogates egress of T cells from lymph nodes led to a decreased number of Id-specific CD4^+^ T cells within the tumor, resulting in failure of tumor rejection (86). Consistent with these findings, the non-secreting MOPC315.37 variant caused little activation of CD4^+^ T cells in draining lymph nodes, and tumor rejection did not occur.

Idiotype-primed APCs are readily found in lymph nodes that drain MOPC315 tumors (Dembic and Bogen, unpublished experiments). It should therefore be possible by cell purifications and characterizations to reveal the identity of these Id-primed APCs in lymph nodes. Information from such experiments could help to define the mechanisms by which APC get primed by secreted tumor antigen. For example, if the predominant features of Id-primed APCs are that of a residential dendritic cell, this may signify priming by soluble antigen arriving to the lymph node via afferent lymphatic vessels.

### Elimination of MHC II^NEG^ tumor cells

It is well documented that Th1/IFN-γ-activated M1 macrophages isolated from tumors under conditions of tumor rejection can directly inhibit the growth of MHC II^NEG^ myeloma cells *in vitro* (34, 36, 39). However, the molecular mechanisms mediating the inhibition of tumor cell growth remain to be established. Possibly, reactive oxygen species could be of importance, since resistance against B16 cells [although in later work reported to be MHC class II^POS^ under conditions of rejection (37)] was reduced in iNOS^−/−^ and NOX2^−/−^ mice (30). Results of Perez-Diez et al. indicate that under some circumstances, NK cells activated by CD4^+^ T cells are important, but the effector mechanisms employed by such NK cells have not been addressed (28).

It is also possible that CD4^+^ T cells could themselves directly kill tumor cells, e.g., through FasL/Fas interactions, similar to what has been described for killing of MHC II^POS^ B lymphoma cells (33), or a perforin/granzyme B-dependent mechanism as described for killing of the MHC II^POS^ B16 cells (37). The efficacy of killing mechanisms of CD4^+^ T cells could also differ for different tumors. Thus, even though Th1 cells efficiently killed transfected A20 cells *in vitro* by a FasL-dependent mechanism, the same cells could not kill MOPC315 (26, 66). Finally, it has been reported that IFN-γ produced by tumor-specific Th1 cells mediate tumor rejection by means of angiostatic effects, thus causing starvation of the tumor (32).

### Do CD4^+^ T cell-mediated immune responses against MHC II^NEG^ tumor cells convey bystander killing of tumor cells that have lost expression of antigen?

In theory, macrophage-mediated killing of MHC II^NEG^ tumors could be expected to indiscriminately kill surrounding cells, including tumor cells that have lost expression of antigen (“bystander killing”). If true, this would be a clinically important asset of Th1/M1 macrophage-mediated killing of tumor cells (34, 36, 39). The previously described angiostatic properties of Th1 derived IFNγ (32) would also be expected to cause bystander killing. On the other hand, direct killing of MHC II^POS^ tumor cells by cytotoxic CD4^+^ T cells was demonstrated not to induce bystander killing (37).

### What CD4^+^ T cell phenotypes support anti-tumor immunity?

Naïve CD4^+^ T cells in Id-specific TCR-transgenic mice, which eradicate injected MHC II^NEG^ tumor cells, develop into IFNγ-secreting Th1 TILs that induce macrophage polarization into tumoricidal M1 macrophages (33, 34, 39). Transfer of naïve Id-specific CD4^+^ T cells could cure established MHC II^POS^ tumors (33). In the Trp1-specific TCR-transgenic model, naïve (37, 38), Th1 (35), and Th17 (35) cells have been demonstrated to eradicate MHC II^POS^ tumors. Collectively, these results indicate that the primary anti-tumor response of naïve CD4^+^ T cells is followed by T cell differentiation into Th1 (or possibly Th17) cells that confer anti-tumor immunity irrespective of MHC class II expression on tumor cells. While Th1 cells are clearly associated with anti-tumor immunity, variable effects have been observed with other CD4^+^ T cell subsets, reviewed in Ref. (90). Moreover, recent studies suggest that effector CD4^+^ T cells retain some degree of functional plasticity (91, 92). The plasticity of effector Th populations may explain the differential effects of the various Th cell populations in tumor immunity. In addition, exploiting the plasticity of Th cell subsets may be utilized in immune therapy.

### Tolerance induction of tumor-specific CD4^+^ T cells

Use of TCR-transgenic mice offers the possibility of studying tolerance development by following the fate and function of tumor-reactive CD4^+^ T cells. When Id-specific TCR-transgenic mice failed to reject high amounts of injected MHC II^NEG^ MOPC315 cells, CD4^+^ T cells in peripheral lymphoid organs and in the tumor became deleted (93). The extent of deletion became more profound as tumor size increased. The deletion of peripheral tumor-specific CD4^+^ T cells seen in this model for a highly secreted tumor antigen resembles that of exhaustion observed in chronic viral diseases. In addition to peripheral deletion of Id-specific CD4^+^ T cells, progressive MOPC315 tumors also caused thymocyte deletion. It was demonstrated that circulatory myeloma protein gained access to the thymus and was presented in an MHC class II context by thymic APCs, thus causing negative selection of thymocytes (94).

In a recent paper, T cell characteristics in Trp1-specific TCR-transgenic mice developing B16 tumor recurrence following adoptive therapy were studied. Recurrence was associated with increased FoxP3^+^ T_reg_ cell numbers, and increased expression of inhibitory ligands, including PD-1 and CTLA-4 inhibitory receptors on both T_reg_ and effector CD4^+^ cells (95). Tumor recurrence could be prevented by concomitant depletion of T_regs_ and administration of checkpoint blockade antibodies. Collectively, these results indicate that CD4^+^ T cells must eliminate tumor antigen-secreting tumor cells efficiently within a short timeframe. If the elimination is incomplete, T cell tolerance is induced by multiple mechanisms.

It has been shown that MHC II^POS^ A20 cells, are not rejected after i.v. injection in HA-specific TCR-transgenic mice, but induce anergy in CD4^+^ T cells via priming of bone marrow derived APCs (89, 96). Interestingly, when presentation by bone marrow derived APCs was prevented by the use of bone marrow chimeras, anergy did not occur, and tumor cells were rejected (72). Thus, it might seem that tumor cells that poorly secrete tumor antigen could favor anergy development by induction tolerogenic APCs. The above results are consistent with previous observations that A20 cells expressing a non-secreted λ2^315^ were not rejected in Id-specific TCR-transgenic mice (26) (although it was not tested if anergy was induced). These results, obtained with non-secreting MHC II^POS^ A20 transfectants in two different TCR-transgenic models, are in support of the notion that tumor-specific antigen, perhaps via presentation of apoptotic or necrotic tumor cells by a special type of APC, favor induction of T cell anergy. In contrast, secretion of tumor-specific antigen and presentation (perhaps by another type of host APCs) in lymph nodes, may favor induction of potent primary anti-tumor CD4^+^ T cell responses.

### Dichotomous role of Th cells in B cell cancers

This review paper has focused on CD4^+^ T cell-mediated eradication of tumor cells. However, CD4^+^ T cells may also induce tumors. This dichotomy may especially apply to B cell tumors since B cells are known to proliferate in response to help from CD4^+^ T cells. Extensive and prolonged B cell proliferation could indeed predispose to genetic instability and malignant transformation. In fact, B lymphoma development has been associated with continuous antigenic exposure in chronic infectious diseases caused by *Helicobacter pylori*, EBV, and hepatitis C. Moreover, chronic immune responses to self antigens in autoimmune diseases such as systemic lupus erythematosus, Sjögren’s syndrome and rheumatoid arthritis have also been linked to development of B cell lymphomas, reviewed in Ref. (97, 98). Further supporting a role for chronic antigen stimulation, diffuse large B cell lymphomas (98, 99) and follicular B cell lymphomas (98, 100, 101) are frequently infiltrated with T cells. In Ig- and TCR-transgenic mice, chronic stimulation of Id^+^ B cells by Id-specific CD4^+^ Th2 cells results in the induction of Id^+^ B lymphomas (102). Moreover, two separate studies have shown that proliferation of B lymphomas (103) and MM (104) was augmented by the presence of CD4^+^ T cells.

The MOPC315 model, reviewed herein, was used in the experiments were Id^+^ lymphomas were induced. Interestingly, when such induced lymphoma cells were injected s.c into naïve Id-specific TCR-transgenic mice, the lymphoma cells were promptly rejected (102). Thus, Id^+^ B lymphoma cells were eliminated by mice having naïve CD4^+^ T cells with an identical Id-specific TCR to that of the B lymphoma-inducing Th2 cells. If naïve T cells in the protected mice differentiated into tumor-eliminating Th1 cells was not investigated. However, analogous experiments indicate that Th1 is the primary response to subcutaneously inoculated B lymphomas (34, 39). These results suggest that B lymphoma cells induced by Th2 cells are rejected by Th1 cells expressing an identical TCR. The finding has obvious implications for T cell therapy: if a B cell tumor is initiated by Th2 cells, it may be treated by Th1 cells of the same specificity (and possibly *vice versa*). The same may apply to other combinations of Th cells such as Th17/Th1 etc. Thus, re-education of T cell phenotype may become part of the tumor immunotherapy armamentarium. Given the plasticity of CD4^+^ subsets (91, 92), such re-education may become a real possibility.

## Concluding Remarks

### How disparate are the mechanisms for rejection of MHC II^POS^ and MHC II^NEG^ tumors?

The data reviewed herein suggest that the difference between direct and indirect killing of tumors relates predominantly to the effector stage of tumor cell killing. Thus, CD4^+^ T cells can kill MHC II^POS^ cells directly, while killing of MHC II^NEG^ occurs indirectly via macrophages or possibly NK cells, angiostatic effects, or all of these. In contrast, the primary activation of naïve tumor-specific CD4^+^ T cells appears to be similar for the direct and indirect mechanisms, in that presentation of tumor-specific antigen by host APC seems to be required. However, the evidence for this in the context of MHC II^POS^ tumors is largely circumstantial. In an MHC II^NEG^ myeloma model, secretion of tumor-specific myeloma protein clearly facilitates priming of APC in lymph nodes and stimulation of naive CD4^+^ T cells that subsequently infiltrate the tumor site. Thus, the nature of the antigen, by virtue of its cellular localization and accessibility to APCs, might determine the ability of the antigen to serve as an efficient tumor-specific antigen in CD4^+^ T cell responses. A more in-depth analysis of such factors might be of value in reconciling observations made in the various TCR-transgenic models.

## Conflict of Interest Statement

The authors declare that the research was conducted in the absence of any commercial or financial relationships that could be construed as a potential conflict of interest.

## Abbreviations

APC, antigen-presenting cell; CIITA, class II trans-activator; FasL, Fas ligand; HA, hemagglutinin; Id, idiotype; IFN-γ, interferon gamma; Ig, immunoglobulin; MHC, major histocompatibility complex; MM, multiple myeloma; MOPC, mineral oil-induced plasmacytoma; OVA, ovalbumin; s.c., subcutaneous; TCR-Tg, T cell receptor transgenic; TIL, tumor-infiltrating lymphocyte; Trp1, tyrosinase-related protein 1; SCID, severe combined immunodeficiency.
